# Development of a nitrogen-doped 2D material for tribological applications in the boundary-lubrication regime

**DOI:** 10.3762/bjnano.8.147

**Published:** 2017-07-17

**Authors:** Shende Rashmi Chandrabhan, Velayudhanpillai Jayan, Somendra Singh Parihar, Sundara Ramaprabhu

**Affiliations:** 1 Department of Physics, Alternative Energy and Nanotechnology Laboratory, Nano Functional Materials Technology Centre, Indian Institute of Technology Madras, Chennai, Tamil Nadu 600036, India; 2 Nano & Applied Coating Material Laboratory, NTPC Energy Technology Research Alliance (NETRA), NTPC Ltd, E3, Ecotech II, Greater Noida 201306, Uttar Pradesh, India

**Keywords:** friction, lubrication, nanolubricant, nitrogen-doped reduced graphene oxide, tribology, wear

## Abstract

The present paper describes a facile synthesis method for nitrogen-doped reduced graphene oxide (N-rGO) and the application of N-rGO as an effective additive for improving the tribological properties of base oil. N-rGO has been characterized by different characterization techniques such as X-ray diffraction, scanning electron microscopy, transmission electron microscopy, X-ray photoelectron spectroscopy and Raman spectroscopy. N-rGO-based nanolubricants are prepared and their tribological properties are studied using a four-ball tester. The nanolubricants show excellent stability over a period of six months and a significant decrease in coefficient of friction (25%) for small amounts of N-rGO (3 mg/L). The improvement in tribological properties can be attributed to the sliding mechanism of N-rGO accompanied by the high mechanical strength of graphene. Further, the nanolubricant is prepared at large scale (700 liter) and field trials are carried out at one NTPC thermal plant in India. The implementation of the nanolubricant in an induced draft (ID) fan results in the remarkable decrease in the power consumption.

## Introduction

Advances in machine technology necessitate the reduction in energy loss by improving the tribological performance. This energy loss is caused primarily by friction and wear. The employment of lubricants in machines reduces friction and wear, which results in energy saving. However, the tribological performance of conventional lubricants (water and oil) fails to meet the demand of newly developed mechanical technologies. Recent development in lubricant technology reveals that the tribological performance of conventional lubricants can be improved by the addition of the solid particles [1–6]. When boundary lubrication occurs the asperities of the sliding surfaces are in direct contact with each other despite the presence of lubricant. Thus, the load is actually carried by the surface asperities [7]. The addition of solid particles is advantageous in the boundary-lubrication regime since the solid particles can move to the surface-contact region and improve the lubrication. This type of lubricants makes use of the ball-bearing mechanism and the high mechanical strength of solid additives.

Several studies have been carried to investigate the tribological performance of lubricant after the addition of solid particles. Initially, most of the studies were concentrated on the carbon C_60_ molecules as additive in lubricant oil [8–10]. Subsequently, researchers studied the tribological properties of carbon-based additives such as graphite [1], graphene [2,6], carbon spheres [11–12] and carbon nanotubes [13–15]. In addition, several reports are available on the tribological properties of nanolubricant based on metals [16], metal oxides [17–18], MoS_2_ [4,19], boron [20] and WS_2_ [21]. Among all the solid additives, 2D graphene is a promising material to improve the tribological performance because of its high surface area to volume ratio and excellent mechanical strength. Lin et al. studied the tribological properties of modified graphene platelets dispersed in oil and shows that the graphene platelets improved the wear resistance and load-carrying capacity of the machine after the modification [22]. Song et al. compared the tribological properties of multiwalled carbon nanotubes and graphene oxide nanosheets as additives for water-based lubricants and found that graphene oxide nanosheets improved the tribological properties more than the carbon nanotubes [23]. Zhang et al. studied the tribological properties of an oil lubricant with oleic acid-modified graphene and obtained 17% and 14% reduction in coefficient of friction (COF) and wear scar diameter (WSD), respectively [24]. Berman et al. studied the friction and wear characteristics of 440C steel test pairs lubricated with graphene and found a remarkable reduction in COF (from ca. 1 to ca. 0.15) and wear [25]. In addition, there are several studies carried out on reduced graphene oxide and revealed that the graphene is the most promising material for enhancing the tribological performance [6,22–23,26–38]. Recently, Jaiswal et al. reported the tribological study on TiO_2_-reinforced boron/nitrogen co-doped graphene oxide and revealed that the doping of reduced graphene oxide with heteroatoms (nitrogen, boron, co-doped nitrogen/boron) successively enhanced the antiwear properties [39]. Considering aforesaid facts, the present work describes the facile synthesis of nitrogen-doped reduced graphene oxide (N-rGO) where the surface of graphene sheet is modified by doping with nitrogen, which results in an excellent stability in base oil. Moreover, the nanolubricants prepared by dispersing a very small amount of N-rGO (3 mg/L) show a significant decrease in the COF (25%) and WSD (15%) and these are the lowest values ever reported for COF for such a small amount of N-rGO. The present work not only describes the tribological performance of the nanolubricant but also reports the application of the developed nanolubricant in an induced draft (ID) fan and thus proves the potential towards commercialization. The implementation of the prepared nanolubricants in the ID fan results a remarkable reduction in the power consumption.

## Experimental

### Synthesis of N-rGO

**Figure 1:**
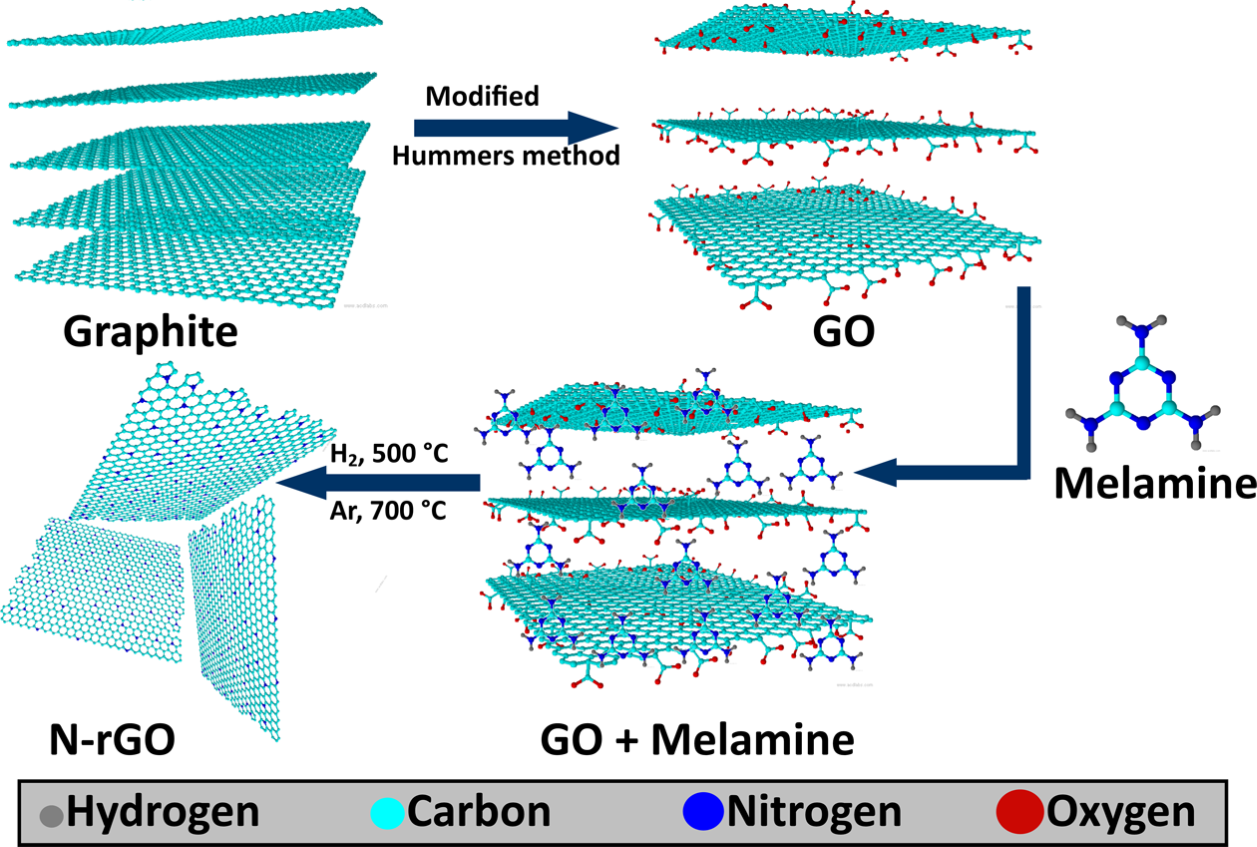
Schematic illustration for the synthesis of N-rGO.

The synthesis of graphene oxide using Hummers method is described in Supporting Information File 1. Nitrogen-doped reduced graphene oxide (N-rGO) was prepared from melamine and GO. Melamine was used as nitrogen precursor. For this, the GO and melamine were mixed thoroughly in 1:1 ratio. The GO and melamine mixture was loaded into a tubular furnace. Initially, argon gas was flushed inside the furnace for 10 min to create an inert atmosphere. Afterwards, the temperature of the furnace was raised to 500 °C and hydrogen gas was flowed through for 30 min. At this temperature, GO was exfoliated into few-layered reduced graphene oxide (r-GO). Further, the temperature of furnace was increased to 700 °C. At this temperature, melamine was decomposed completely resulting in the uniform doping of nitrogen in graphene. The final sample is labeled as N-rGO. Figure 1 shows the schematic for the synthesis of N-rGO.

### Characterization techniques

The powder X-ray diffraction (XRD) patterns were recorded in the range of 2θ = 5° to 2θ = 90° using a Rigaku X-ray diffractometer. Raman scattering spectra of graphite, GO and N-rGO were collected by using a WITec Raman spectrometer equipped with Nd:YAG laser (λ = 532 nm). The surface morphology of the sample was analyzed by using field-emission scanning electron microscopy (FEI quanta FEG200). Transmission electron microscopy images were taken using transmission electron microscope (JEOL JEM-2010F) operated at 200 keV. X-ray photoelectron spectroscopy was performed to confirm the presence of various elements. Nanolubricants were prepared by using a probe sonicator (Sonics, 500 W).

#### Tribological testing

The tribological properties of nanolubricants were studied using a four-ball tester (Magnum Engineers, Bengaluru, India). The test balls are made of chrome steel alloy having a diameter of 12.7 mm according to ANSI standard. The measurements were performed according to ASTM 5183 standard.

#### Nanolubricant preparation

**Figure 2:**
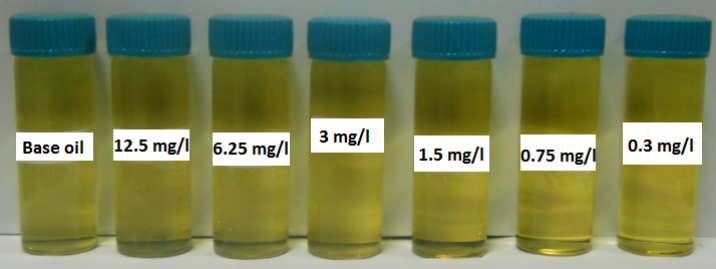
N-rGO dispersion nanolubricant at different concentration.

Nanolubricants were prepared by dispersing N-rGO in oil using probe sonication. The probe sonication was done for 30 min with a 20 kHz frequency and a power of 500 W (20%) using probe sonicator. Different nanolubricants with different amounts of N-rGO were prepared and the stability of these nanolubricants was checked by probing the settlement after one-month time. Figure 2 shows the photographs of nanolubricants prepared in base oil with different amounts of N-rGO with a minimum of 3 mg/L and a maximum of 12.5 mg/L. The N-rGO lubricants have a good stability as seen in Figure 2.

#### Friction and wear test

**Figure 3:**
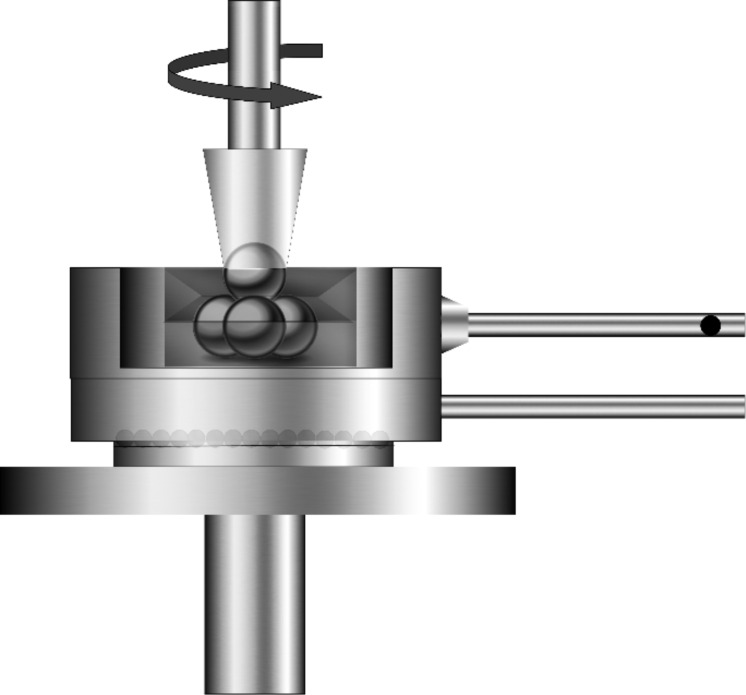
Schematic of the ball-pot assembly in a four-ball tester.

The COF and wear test of N-rGO nanolubricant was studied by using a four-ball tester. In a four-ball tribotester, an upper ball rotates with a specific speed against the three stationary balls at an applied load. This rotation results in the friction and wears at the contact points of the three stationary balls. For the present study, the friction and wear tests were carried out at a rotating speed of 600 rpm by applying a constant load of 400 N. The tests were carried out for 60 min at 75 °C. Before carrying out the measurements, the four test balls were ultrasonicated in ethanol for 10 min and dried. The other components were cleaned with ethanol and the cleaned three test balls were clamped together in the ball-pot assembly. The test balls were covered with nanolubricant for measurements. The fourth ball was clamped in the upper holder and the constant load of 400 N was applied. Figure 3 represents the schematic of the ball-pot assembly in a four-ball tester.

## Results and Discussion

### Materials characterization

The XRD patterns of graphite, GO and N-rGO are depicted in Figure S1 (Supporting Information File 1). The intense crystalline peak of graphite occurs at ca. 26°, which is the characteristic peak of hexagonal graphite with a *d*-spacing of 0.34 nm. Upon conversion of graphite into GO, the peak position shifts to ca. 11° corresponding to an interlayer spacing of 0.79 nm. This increase in *d*-spacing can be attributed to the intercalation of –OH and –COOH functional groups in the graphite layers. N-rGO shows a broad peak from ca. 15° to 36° with a *d*-spacing of 0.37 nm. The decrease in the interlayer spacing from 0.79 to 0.37 nm suggests the removal of oxygen-containing functional groups from the GO interlayer during exfoliation [40]. This broad peak is also indicative of a loss of the long-range order in graphene.

Raman spectroscopic measurements (Figure S2, Supporting Information File 1) were performed at low laser intensities in order to avoid the burning of samples. A highly intense peak is observed for graphite at 1589 cm^−1^ corresponding to the G band. The lack of D band peaks in graphite implies that the graphite is defect-free. A shift of the G band of GO located at 1610 cm^−1^ is observed [40]. However, after reduction and nitrogen doping, the peak corresponding to the G band shifted back to 1589 cm^−1^, close to the value of graphite. The presence of the D band, with an intensity comparable to that of the G band, in case of GO and N-rGO suggests that defects are created during the chemical treatment.

SEM and TEM images of the N-rGO are shown in Figure S3 (Supporting Information File 1). N-rGO exhibits a wrinkled surface, which can be attributed to the rapid removal of functional groups during exfoliation. The size of N-rGO sheets is of a few square micrometers. The samples for TEM analysis are prepared by dispersing small amount of N-rGO in 2-propanol. The dispersion is ultrasonicated for 10 min and then drop-cast over a carbon-coated copper grid (200 mesh). TEM images shown in Figure S3c,d (Supporting Information File 1) reveal that N-rGO has few layers and shows folding at some places.

In order to confirm the nitrogen doping in N-rGO, X-ray photoelectron spectroscopy was carried out. The XPS survey spectrum of N-rGO confirms the presence of carbon (87.5 atom %), oxygen (8.9 atom %) and nitrogen (3.6 atom %) (Figure S4, Supporting Information File 1). The high-resolution C 1s spectra show three components corresponding to sp^2^ C=C bonds (at 284.6 eV), sp^2^ C=N bonds (at 285.9 eV) and sp^3^ C–N bonds (at 288.8 eV). The peaks at 285.9 and 288.8 eV can be assigned to oxygen-containing functional groups, i.e., C=O and C–OH, respectively [41]. The high-resolution N 1s spectrum of N-rGO is deconvoluted into three peaks corresponding to graphitic N (at 401.1 eV), pyridinic N (at 398.2 eV) and pyrrolic N (at 400.1 eV).

### Optimization of additive

**Figure 4:**
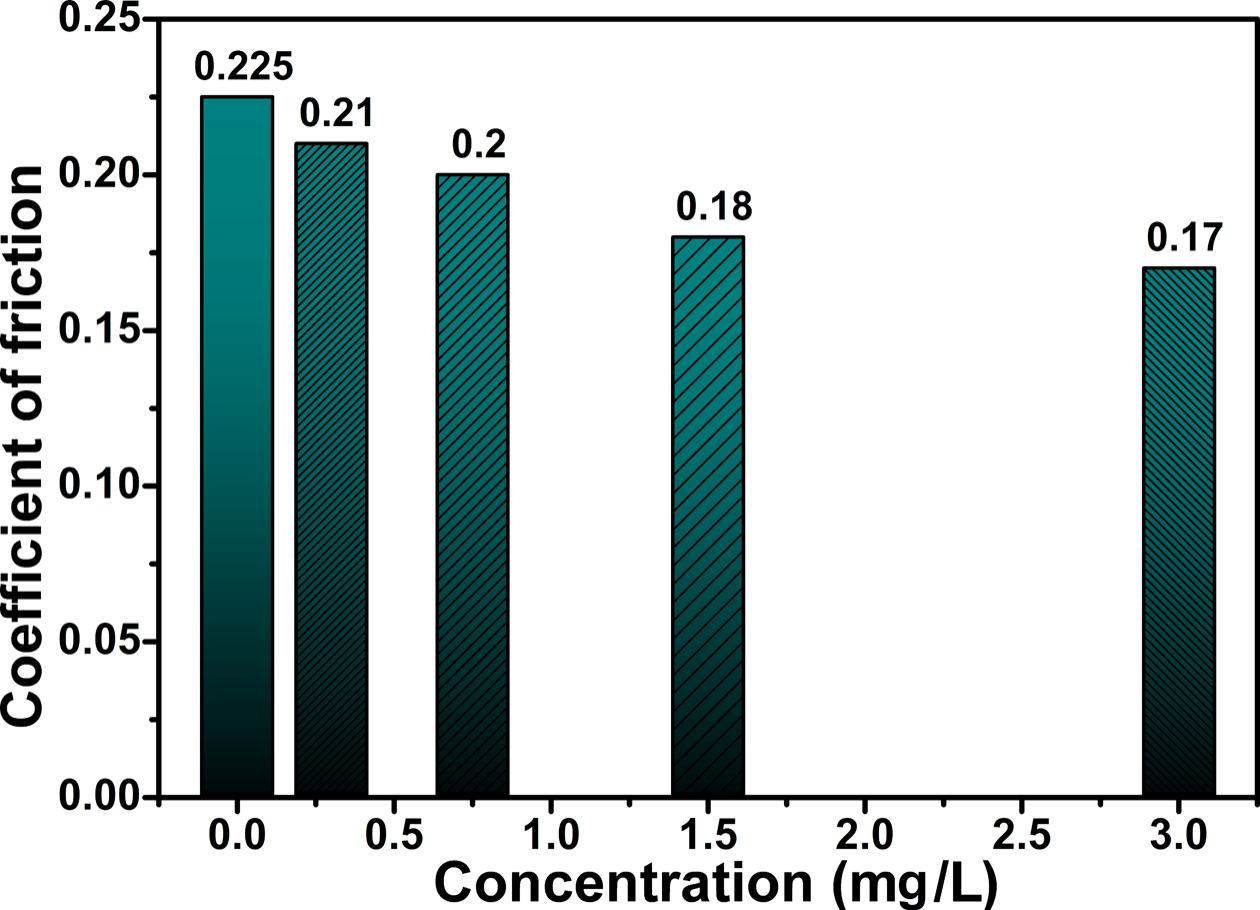
Coefficient of friction at low N-rGO concentrations.

**Figure 5:**
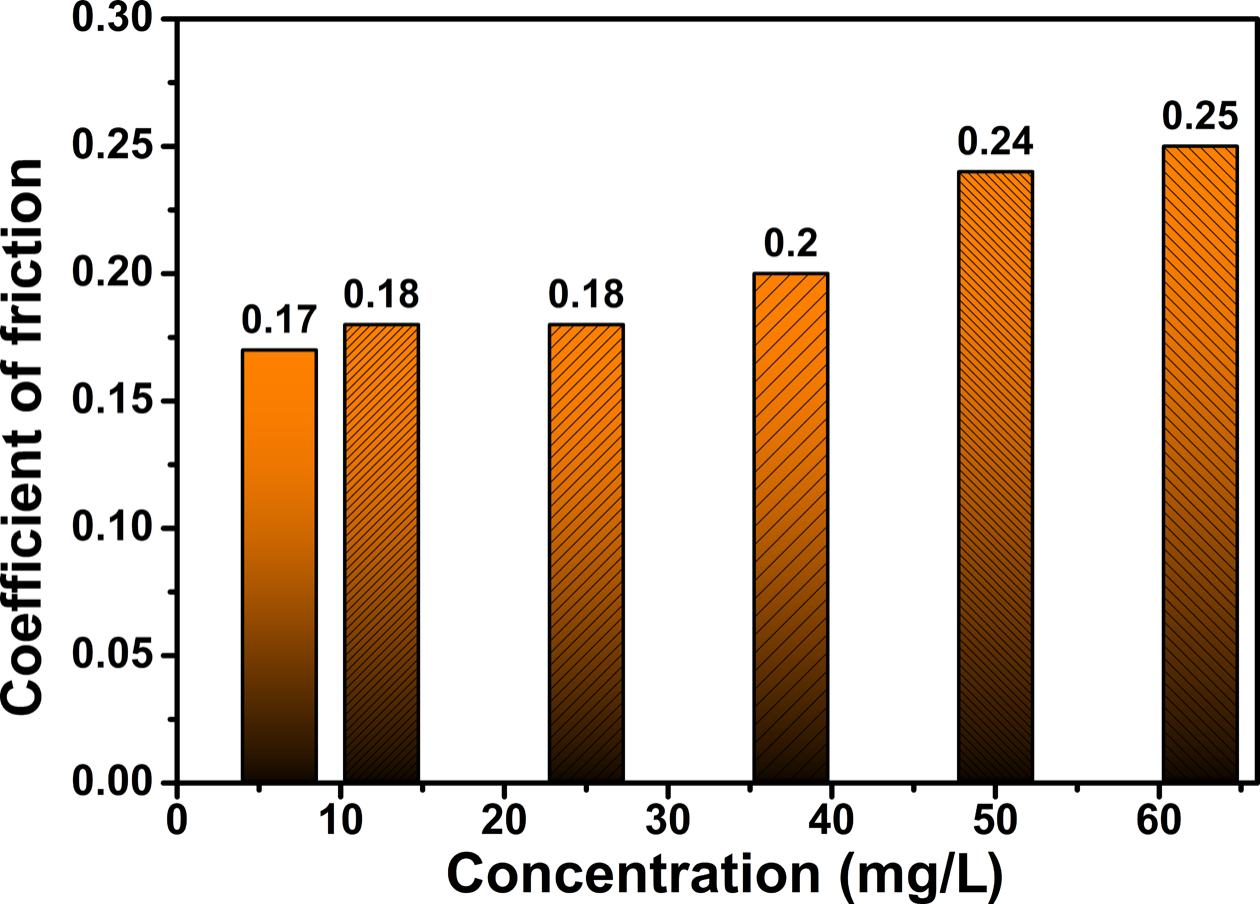
Coefficient of friction at high N-rGO concentrations.

In order to optimize the concentration of additive, the nanolubricants were prepared with various concentrations and tested their tribological properties. Figure 4 and Figure 5 give the change in the COF as a function of the concentration of additive (N-rGO) at the ASTM standard. One can see that the optimum amount of the additive (N-rGO) is 3 mg/L for which the maximum decrease of COF was obtained. COF decreases from 0.225 (base oil) to 0.170 (3 mg/L of N-rGO), which is a reduction of about 25%. The excessive additive in base oil increases the COF of nano-lubricant (Figure 5). For comparison, the COF at higher concentrations of N-rGO is shown in Figure 5. The COF increases with increasing concentration of N-rGO additive. This increase can be attributed to the formation of aggregates between the contact surfaces resulting in poor lubrication. These measurements suggest that the optimum amount of additive is 3 mg/L, which is the concentration used in all further experiments.

**Figure 6:**
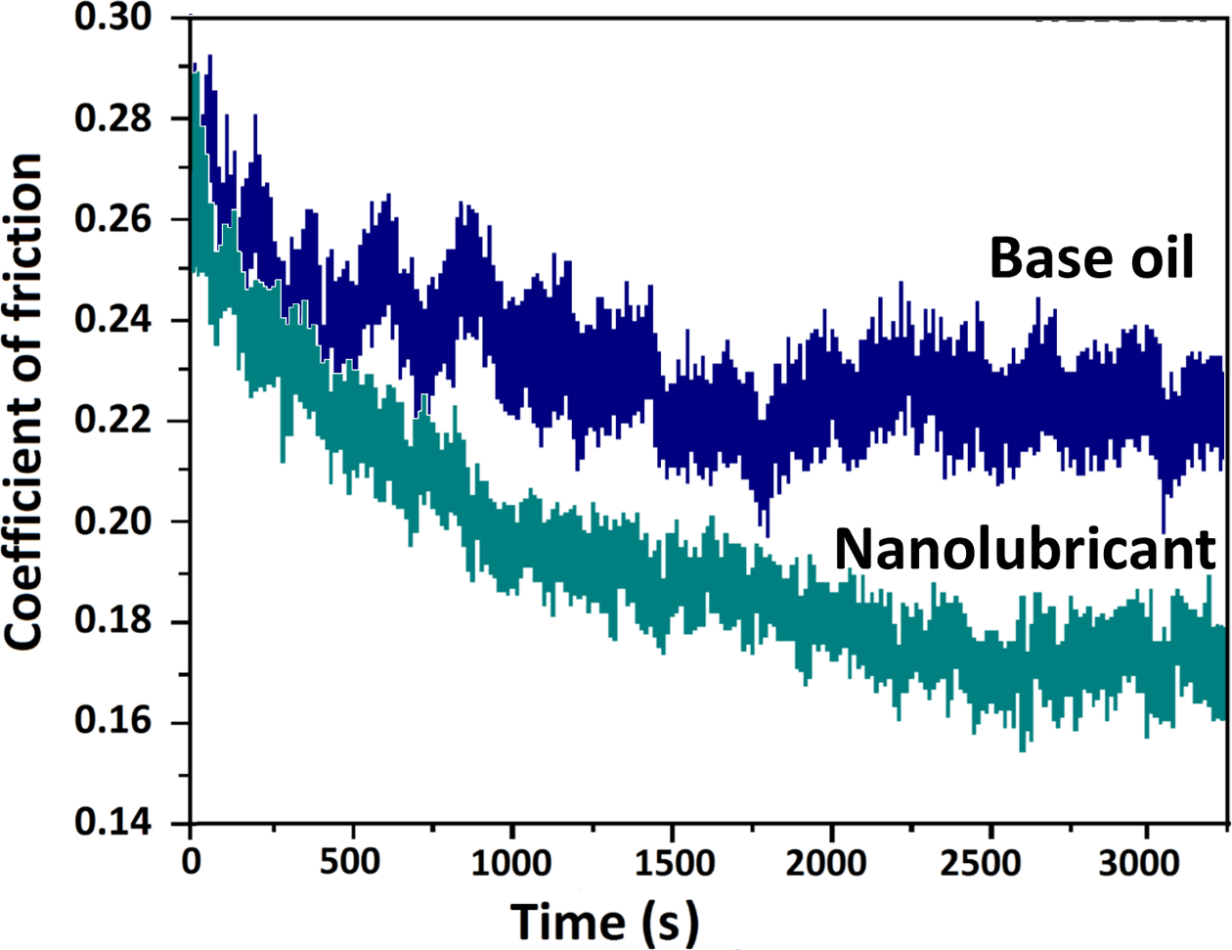
Variation of COF over time for base oil and nanolubricant with N-rGO (3 mg/L).

Figure 6 shows the measurements of COF with base oil and the nanolubricant with N-rGO (3 mg/L). The COF of nanolubricant is lower than that of base oil. For 2D nanostructures, the improvement in tribological properties is well understood as sliding mechanism. The significant decrease in the COF for N-rGO dispersed nanolubricant can be ascribed to the layered morphology of N-rGO, which allows the easy intrusion in the friction interfaces. In the boundary-lubrication regime, friction surfaces are in direct contact with each other and the presence of N-rGO sheets between the moving contact surfaces avoids the direct contact and thus reduces the COF.

**Figure 7:**
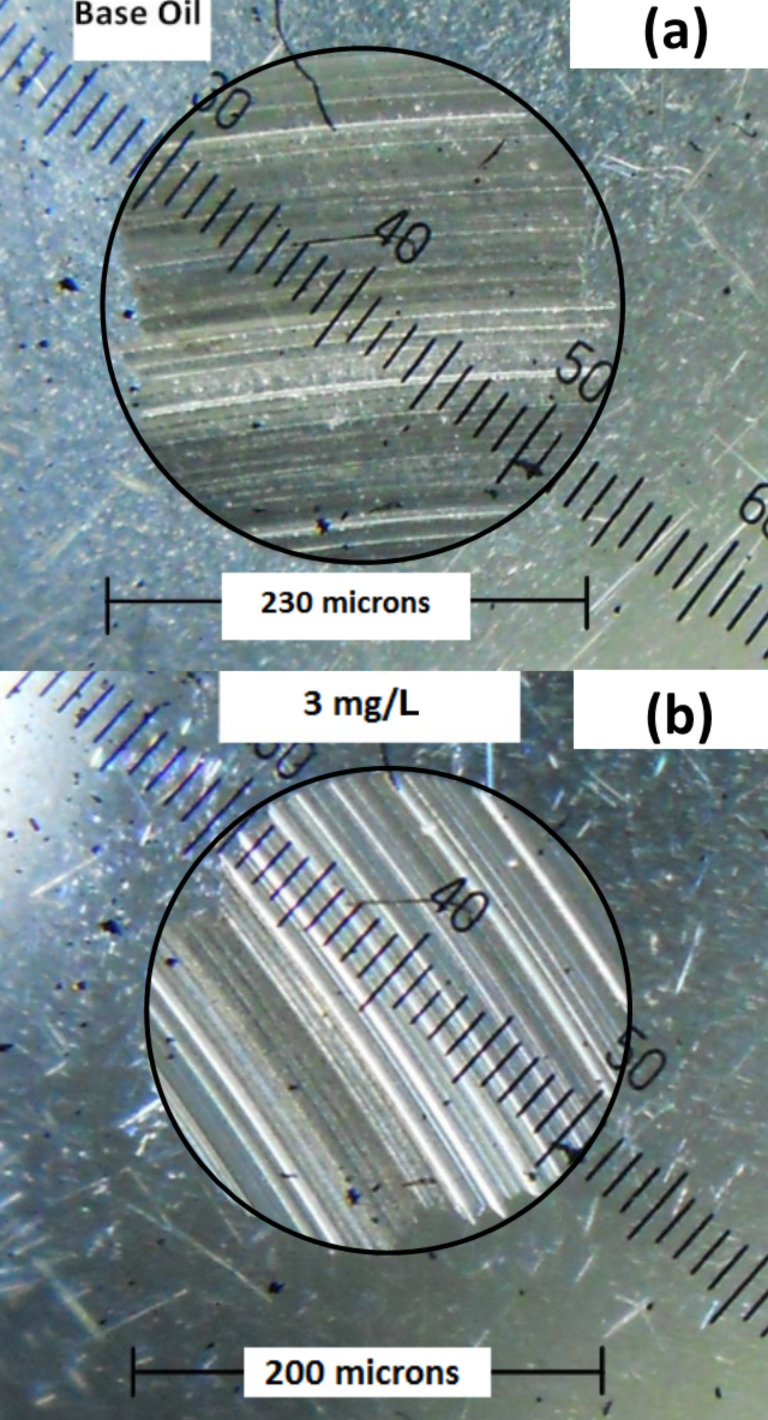
Wear scar diameter (WSD) of stainless steel balls lubricated with (a) base oil and (b) N-rGO nanolubricant (3 mg/L).

WSD of the test ball with base oil and N-rGO nanolubricant is shown in Figure 7. With N-rGO (3 mg/L) oil the WSD is reduced to 200 μm from 230 μm for base oil (15% reduction). The significant decrease in WSD can be attributed to the easy penetration of N-rGO sheets into the contact points [2,39], which reduces the direct contact between the rough surfaces.

**Figure 8:**
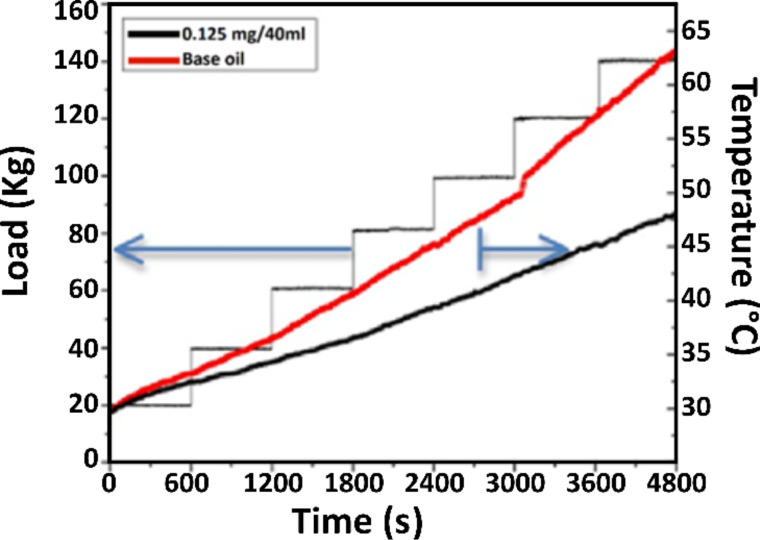
Comparison of the measured temperature of base oil and N-rGO (3 mg/L) nanolubricant as a function of the load.

The measured temperature as a function of the gradually increased normal load during the experiment is plotted and compared in Figure 8 for base oil and N-rGO oil. The temperature increased to 62.5 °C for base oil while for N-rGO nanolubricant, the temperature increased to 47.5 °C at 1400 N. This indicates that the developed N-rGO based nanolubricant not only reduces the COF, but also acts as heat transfer fluid and decreases the temperature at friction interfaces.

### Application of the nanolubricant in induced draft (ID) fans

**Figure 9:**
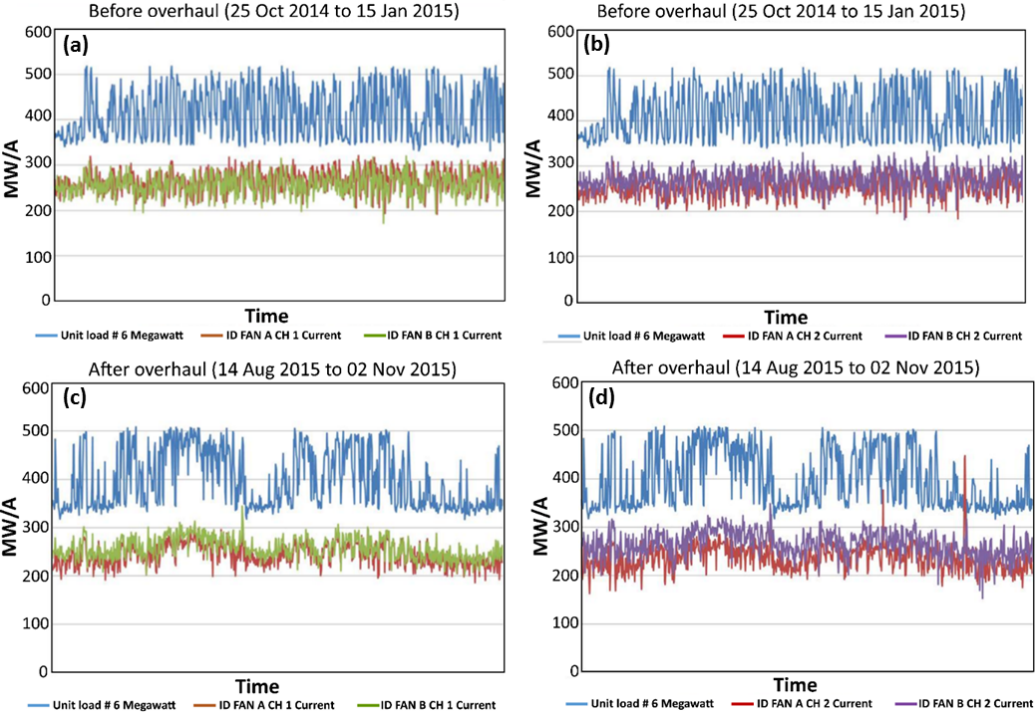
Unit load and current of ID fan A and B for (a) Ch 1, (b) Ch 2 before overhaul (replacement of base oil with nanolubricant), (c) Ch 1 and (d) Ch 2 after overhaul.

Stable nanolubricants were prepared in large scale (700 liter) for field trial at a power plant of the National Thermal Power Corporation (NTPC) in India. The study was performed on two different induced draft (ID) fans (A and B) on two different channels (Ch 1 and Ch 2) using an energy management system. Initially, the data were recorded for 80 days at an interval of 1 h before the base oil was replaced with the nanolubricant during an overhaul. Figure 9a,b show the variation of unit load and current in fan A and fan B for Ch 1 and Ch 2 with base oil. Figure 9a shows that the average current in Fan A and Fan B (Ch 1) is about 255 A. From Figure 9b, it is observed that the current in fan A and fan B (in Ch 2) is not exactly equal. The average value of the current of fan A turned out to be 245 A whereas the average value of the current of fan B was 265 A.

The lubricant in fan A was then replaced with the N-rGO nanolubricant while the lubricant in ID fan B remained base oil in both channels. Figure 9c,d show the variation of unit load and current in Ch 1 and Ch 2 after replacing the lubricant in fan A. It is clear that the current in fan A is lower than current in fan B. For Ch 1, the average value of current for fan A and fan B was 250 A and 265 A. Similarly for Ch 2, the average value of current for fan A was 240 A whereas for fan B it was 275 A. Thus, the difference in current between fan A and fan B was increased from 20 A to 35 A in Ch 2.

In summary, before the overhaul for Ch 1 there was no difference in current but after deployment of N-rGO nanolubricant the difference observed was 15 A. In case of Ch 2, before overhaul the difference was 20 Amp, which increased to 35 Amp after the implementation of nanolubricant. So, in conclusion the average difference due to the application of nanolubricant is 15 A for both channels.

**Figure 10:**
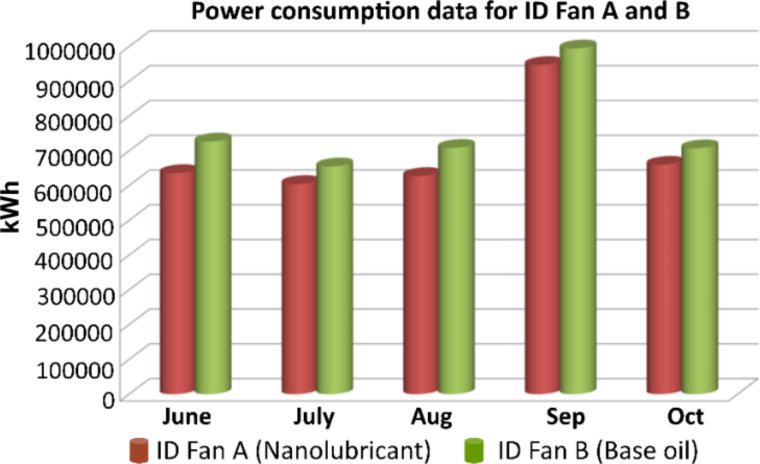
Power Consumption data for ID Fan A and B for few months.

Table 1 shows the power consumed by fan A and fan B for each month. It is very clear that the power consumed by fan A was considerably lower than that consumed by fan B for both channels. Figure 10 shows the total power consumption of fan A (Ch 1 and 2) and fan B (Ch 1 and 2).

**Table 1:** Power consumption of ID fans A and B over several months.

	June (kWh)	July (kWh)	Aug (kWh)	Sep (kWh)	Oct (kWh)

ID fan A (Ch 1)	309457.92	294362.11	305819.70	461072.16	318319.49
ID fan A (Ch 2)	326387.20	310225.23	321657.49	485770.78	341571.97
ID Fan B (Ch 1)	366251.52	330099.71	357024.60	501537.57	356346.24
ID fan B (Ch 2)	360467.45	324949.28	351382.42	492960.35	350942.59
ID fan A (Ch 1 + Ch 2)	635845.12	604587.34	627477.19	946842.94	659891.46
ID fan B (Ch 1 + Ch 2)	726718.98	655048.99	708407.02	994497.92	707288.83
difference (B − A)	90873.856	50461.651	80929.829	47654.976	47397.376

## Conclusion

The present work describes the synthesis of N-rGO and a nanolubricant prepared by dispersing different amounts of nanoparticles in base oil. The study of tribological properties shows the significant decrease in COF when the nanolubricant is used. The measurement of COF was carried out for different concentrations of N-rGO and the maximum decrease in COF (25%) was obtained for a concentration of 3 mg/L. Moreover, the WSD for nanolubricant was reduced considerably compared to the base oil. The field study of nanolubricant in ID fans shows that there is remarkable decrease in the power consumption after the application of the nanolubricant. Thus, N-rGO nanolubricants are very promising for the tribological application due to the considerable improvement of tribological properties.

## Supporting Information

File 1 Additional experimental data.
