# Supplementary material for: Development of a nitrogen-doped 2D material for tribological applications in the boundary-lubrication regime
Source: Beilstein J Nanotechnol. 2017 Jul 17;8:1476–83. doi: 10.3762/bjnano.8.147 (PMC5530610; doi:10.3762/bjnano.8.147)
Supplement: File 1 — Additional experimental data. [file Beilstein_J_Nanotechnol-08-1476-s001.pdf]

Supporting Information

for

Development of a nitrogen-doped 2D material for tribological applications in the boundary-lubrication regime

Shende Rashmi Chandrabhan[1], Velayudhanpillai Jayan[2], Somendra Singh Parihar[2] and Sundara Ramaprabhu*,[1]

Address: [1]Department of Physics, Alternative Energy and Nanotechnology Laboratory, Nano Functional Materials Technology Centre, Indian Institute of Technology Madras, Chennai, Tamil Nadu- 600036, India and [2]Nano & Applied Coating Material Laboratory, NTPC Energy Technology Research Alliance (NETRA), NTPC Ltd, E3, Ecotech II, Greater Noida-201306 Uttar Pradesh, India

Email: Sundara Ramaprabhu* - ramp@iitm.ac.in

[*] Corresponding author

Additional experimental data

Synthesis of graphene oxide (GO)

Lately, graphene has emerged as an ideal material for different energy applications because of its high surface area to volume ratio, good mechanical strength and excellent electronic and thermal conductivity. To obtain a high yield of graphene sheets for various applications, graphene sheets were synthesized by chemical reduction of graphene oxide (GO). First, GO was prepared from natural graphite using Hummers method [1]. In a typical synthesis, graphite powder (2 g) was added to conc. H_2SO_4 (46 mL) while being constantly stirred on an ice bath. Following, 1 g of $NaNO_3$ and 6 g of $KMnO_4$ were added gradually and successively. The suspension was removed from the ice bath and allowed to cool to room temperature while being stirred. Subsequently, DI water (92 mL) was added to the mixture. After 15 min, warm DI water (280 mL) was added to dilute the reaction mixture. Afterwards, 12 mL of H_2O_2 was added until the solution turned brightly yellow. The residue was diluted using water and the resulting suspension was filtered. The final product was dried under vacuum at 60 °C. Here, 2 g of graphite results in ca. 4 g of GO.

Characterization of N-rGO

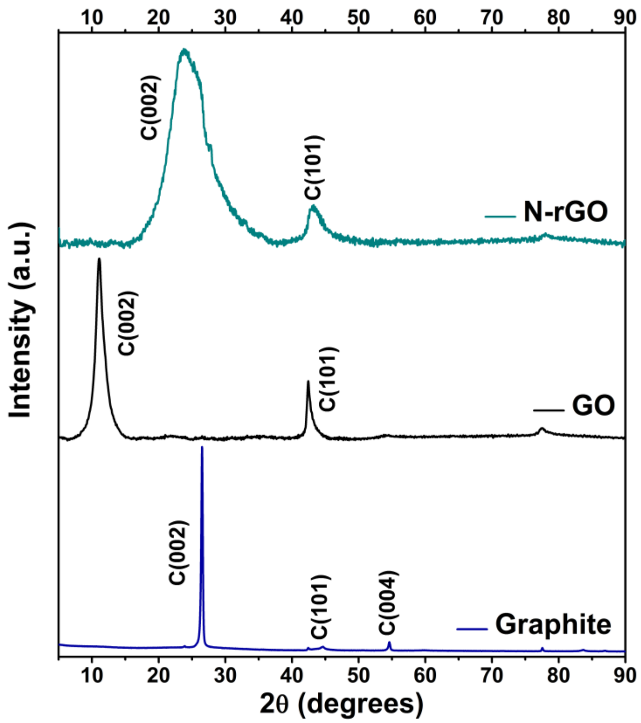

Figure S1: Powder X-ray diffraction pattern of graphite, GO, and N-rGO.

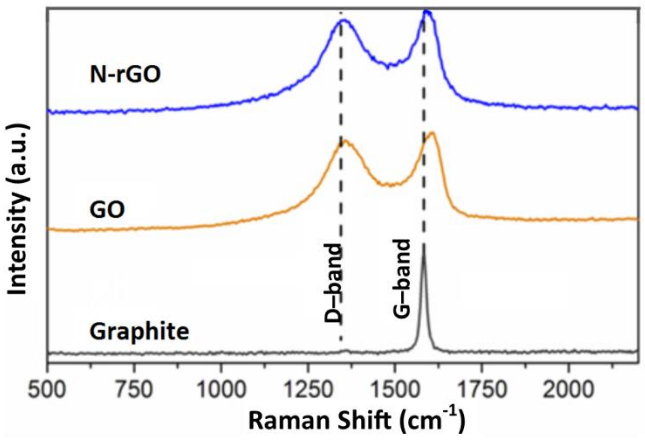

Figure S2: Raman spectra for Graphite, GO and N-rGO.

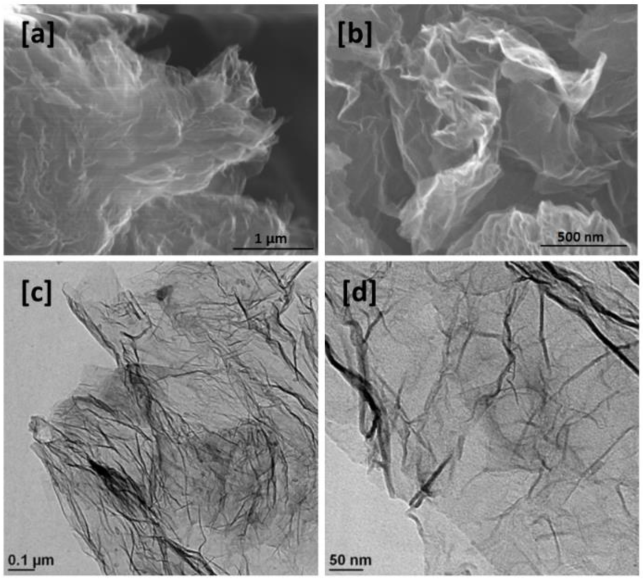

Figure S3: (a, b) SEM images and (c, d) TEM images of N-rGO at different resolutions.

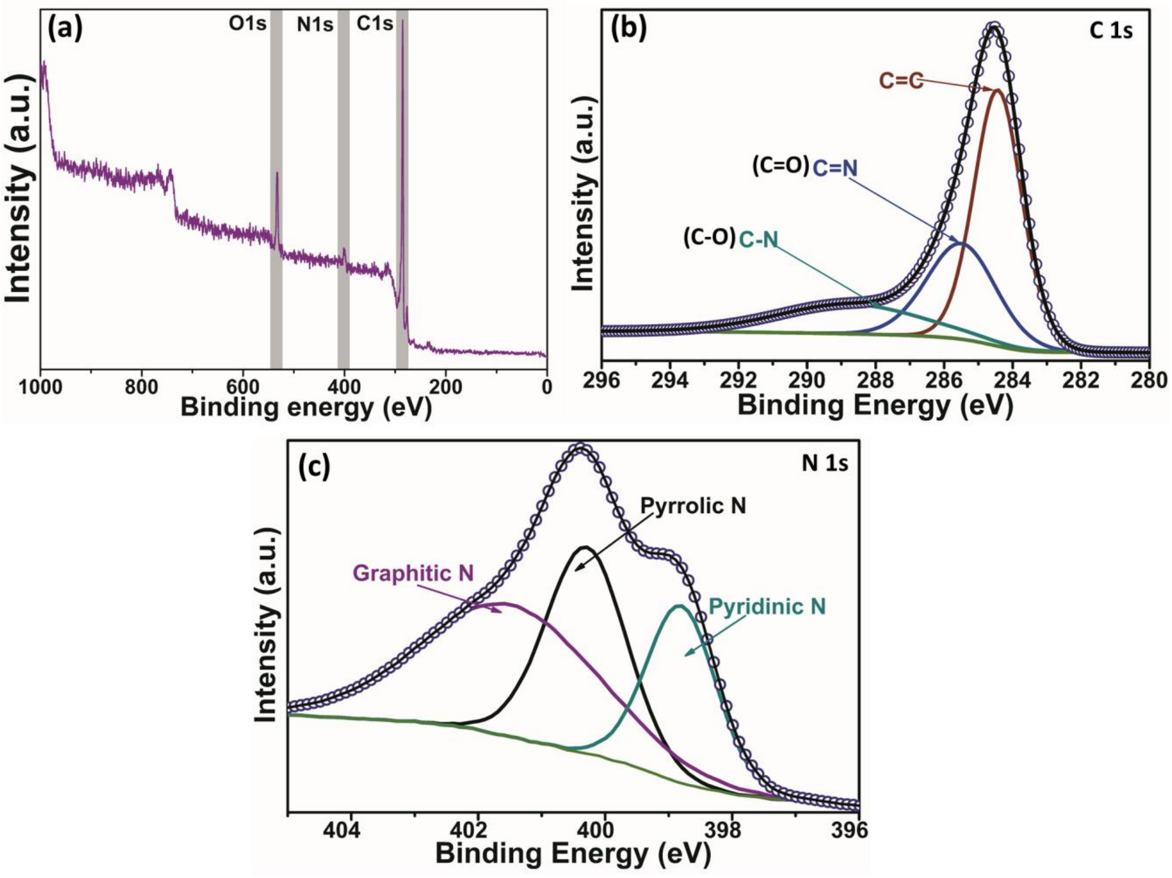

Figure S4: XPS (a) Survey spectra, (b) C 1s spectra and (c) N 1s spectra of N-rGO.

References

1. Hummers, W. S.; Offeman, R. E. *J. Am. Chem. Soc.* **1958,** *80*, 1339. doi:10.1021/ja01539a017
